# Akt-Induced Phosphorylation of N-CoR at Serine 1450 Contributes to Its Misfolded Conformational Dependent Loss (MCDL) in Acute Myeloid Leukemia of the M5 Subtype

**DOI:** 10.1371/journal.pone.0070891

**Published:** 2013-08-05

**Authors:** Dawn Sijin Nin, Azhar Bin Ali, Koichi Okumura, Norio Asou, Chien-Shing Chen, Wee Joo Chng, Matiullah Khan

**Affiliations:** 1 Cancer Science Institute of Singapore, Yong Loo Lin School of Medicine, National University of Singapore, Singapore; 2 Department of Medicine, Yong Loo Lin School of Medicine, National University of Singapore, Singapore; 3 Department of Biochemistry, Yong Loo Lin School of Medicine, National University of Singapore, Singapore; 4 Department of Haematology, Kumamoto University, Kumamoto, Japan; 5 Division of Hematologyand Oncology, School of Medicine, Loma Linda University, Loma Linda, California, United States of America; 6 Department of Haematology-Oncology, National Cancer Institute of Singapore, National University Health System, Singapore; 7 School of Medicine, Asian Institute of Medicine, Science and Technology (AIMST), Bedong, Malaysia; Ohio State University Comprehensive Cancer Center, United States of America

## Abstract

The nuclear receptor co-repressor (N-CoR) is a key component of the generic co-repressor complex that plays an important role in the control of cellular growth and differentiation. As shown by us recently, the growth suppressive function of N-CoR largely relies on its capacity to repress *Flt3*, a key regulator of cellular gorwth during normal and malignant hematopoesis. We further demonstrated how de-repression of *Flt3* due to the misfolded conformation dependent loss (MCDL) of N-CoR contributed to malignant growth in acute myeloid leukemia (AML). However, the molecular mechanism underlying the MCDL of N-CoR and its implication in AML pathogenesis is not fully understood. Here, we report that Akt-induced phosphorylation of N-CoR at the consensus Akt motif is crucial for its misfolding and subsequent loss in AML (AML-M5). N-CoR displayed significantly higher level of serine specific phosphorylation in almost all AML-M5 derived cells and was subjected to processing by AML-M5 specific aberrant protease activity. To identify the kinase linked to N-CoR phosphorylation, a library of activated kinases was screened with the extracts of AML cells; leading to the identification of Akt as the putative kinase linked to N-CoR phosphorylation. Consistent with this finding, a constitutively active Akt consistently phosphorylated N-CoR leading to its misfolding; while the therapeutic and genetic ablation of Akt largely abrogated the MCDL of N-CoR in AML-M5 cells. Site directed mutagenic analysis of N-CoR identified serine 1450 as the crucial residue whose phosphorylation by Akt was essential for the misfolding and loss of N-CoR protein. Moreover, Akt-induced phosphorylation of N-CoR contributed to the de-repression of *Flt3*, suggesting a cross talk between Akt signaling and N-CoR misfolding pathway in the pathogenesis of AML-M5. The N-CoR misfolding pathway could be the common downstream thread of pleiotropic Akt signaling activated by various oncogenic insults in some subtypes of leukemia and solid tumors.

## Introduction

Balanced transcriptional control maintained by the coordinated actions of co-activator and co-repressor proteins and sequence specific transcriptional factors play an important role in the normal growth and development of cells in the hematopoietic system [1]. When mutated or deregulated, these co-activators and co-repressors become key initiators of malignant changes in acute myeloid leukemia (AML; [2], [3]). Nuclear receptor co-repressor (N-CoR) is a key component of the generic co-repressor complex essential for transcriptional repression. First identified as a co-repressor of the un-liganded nuclear hormone receptors RAR (retinoic acid receptor) and TR (thyroid hormone receptor; N-CoR was subsequently shown to be essential for the transcriptional repression mediated by tumor suppressor Mad and other sequence-specific transcription factors [4]–[7]. In our previous studies, we identified N-CoR as a key component of the multi-protein repressor complex containing Ski, PML and HDAC proteins, and defined the crucial role of N-CoR in transcriptional repression mediated by the tumor suppressors Mad and Rb [8]–[10]. More recently, we identified an important role for N-CoR in the repression of *Flt3*, a key regulator of cellular growth during normal and malignant hematopoesis [11]. We further demonstrated how deregulation of *Flt3* due to a misfolded conformation dependent loss (MCDL) of N-CoR contributed to the malignant growth and transformation of cells in acute myeloid leukemia of the FAB-M5 subtype (AML-M5) [11]–[14]. Recently, loss of N-CoR function was linked to the activation of Akt dependent survival pathway in thyroid cancer cells [15]. Moreover, Akt-induced phosphorylation of N-CoR contributed to its cytosolic export in cytokine stimulated neuronal stem cells, suggesting that N-CoR function could be adversely affected by Akt [16]. Aberrant Akt activation through its phosphorylation has been implicated in the pathogenesis of many human tumors, including AML [17]. In a recent report, selective Akt activation was observed in multiple human primary AML-M5 cells but not in normal cells surrounding the malignant tissue, suggesting a key role of Akt in the pathogenesis of AML-M5 [18].

Akt is a serine/threonine kinase which plays an important regulatory role in multiple cellular processes including transcription, cell proliferation and migration. Akt’s role in transcription was first suggested by the finding that growth factors could trigger the nuclear translocation of Akt1 and Akt2 by inducing their detachment from the cell membrane [19], [20]. Later, a crucial role of Akt in the transcriptional control mediated by the Forkhead family of transcription factors, including FKHR, FKHRL1/AF6q21 and AFX, was identified [21]–[30]. Akt was thought to modulate the function of these transcription factors mainly by regulating their subcellular distribution [31], [32]. Akt-induced phosphorylation of FKHR and FKHRL1 promoted their cytosolic retention, eventually sequestering them away from their nuclear targets. Akt also inhibited the function of transcription factor GATA2 through similar mechanism [33]. These findings suggested that an activated Akt could contribute to malignant growth and transformation by modulating the function of key transcription factors involved in cellular differentiation and growth.

AML-M5, also known as acute monoblastic or monocytic leukemia, is a group of malignant disorder characterized by the abnormal accumulation of immature cells of myelo-monocytic lineage in the bone marrow and peripheral blood [34], [35]. AML-M5, which represents 5 to 10% of all AML in human adults, is caused primarily by an array of genetic defects including chromosomal translocation involving various genes. Despite the varied genetic backgrounds, leukemic cells in all AML-M5 variants display an almost identical phenotype characterized by their differentiation arrest and increased proliferative potential. How these diverse genetic anomalies linked to AML-M5 pathogenesis create an almost uniform morphological feature in AML-M5 variants is largely unknown. Our recent work demonstrated that loss of N-CoR mediated transcriptional control of *Flt3* due to the misfolding of N-CoR partly contributed to the malignant growth and transformation of cells in AML-M5 [11]. Given the uniform loss of N-CoR in all AML-M5 variants, we hypothesized that N-CoR misfolding might be a key factor in AML pathogenesis and therefore set out to identify the potential kinase responsible for the misfolding and loss of N-CoR in AML-M5. Here, we report that Akt-induced phosphorylation of N-CoR at serine 1450 contributed to its misfolding and loss in AML-M5 derived cells of varied genetic background, ultimately leading to the abrogation of N-CoR function and eventual deregulation of genes normally repressed by N-CoR. The nearly identical loss of misfolded N-CoR across AML-M5 cells of varied genetic background suggested that misfolded N-CoR could actually be the common pathogenic factor involved in the transformation of cells in different AML-M5 variants.

## Materials and Methods

### AML Cell Lines, Primary AML Samples and Reagents

The AML-M5 cell lines THP-1, Mono-Mac-1 (MM1), Nomo-1 and M-V4–11; the non-AML-M5 cell lines HL-60, U937 and K562; and the APL cell line NB4 were maintained in RPMI 1640 medium (Life Technologies, Gaithersburg, MD) supplemented with 10% Fetal Bovine Serum (FBS; Hyclone Laboratories, Logan, UT). The AML-M5 cell line SigM5 was maintained in Isocove’s modified medium (Life Technologies, Gaithersburg, MD) supplemented with 20% FBS while 293T cells were maintained in DMEM (Sigma Aldrich, MO, USA) enriched with 10% FBS. These cell lines were purchased from ATCC (Manassas, VA, USA), DSMZ - Deutsche Sammlung von Mikroorganismen und Zellkulturen GmbH (German Collection of Microorganisms and Cell Cultures) Germany and Japan Health Sciences Foundation (Osaka, Japan). Primary leukemic samples used in this study were obtained at the time of diagnosis with written informed consent obtained from the patients in accordance with the declaration of Helsinki. Diagnoses of AML were made based on the morphology and cytochemistry according to the French–American–British (FAB) classification. This study was approved by the Institutional Review Boards of National University of Singapore and Kumamoto University, Japan. The N-CoR (C-20) (goat polyclonal) antibody was purchased from Santa Cruz Biotechnology (CA, USA) and used as described previously [12]–[14]. Akt, phospho-Akt (Ser473) and phospho-Akt substrate (RXRXX pS/pT) antibodies were purchased from Cell Signaling Technologies (MA, USA). N-CoR stabilizing agents 4-(2-Aminoethyl) benzenesulfonyl fluoride hydrochloride (AEBSF) and Genistein were used as described previously [13], [14]. Akti-X (Merck, Darmstadt, Germany) was used as described elsewhere [36], [37].

### Protein Solubility Assay

293T cells were transfected using Fugene 6 (Roche, Germany) with plasmid pAct-N-CoR-Flag in combination with constitutively active myristoylated Akt (pGFP-myr-Akt) plasmid or Empty vector. N-CoR solubility assay was performed as described previously [13], [14]. Briefly, Cellular extracts were prepared in NET buffer (20 mM Tris pH8.0, 300 mM NaCl, 1 mM EDTA, 0.5% NP-40, 1 tablet/10 ml Complete Mini protease inhibitor tablet [Roche, Germany]) followed by high speed centrifugation at 20000 g for 10 minutes to separate the soluble and insoluble fractions. Level of N-CoR protein in each fraction was then analyzed by western blotting using Flag antibody (Sigma, CA, USA). Solubility of N-CoR stabilized by various agents in THP-1 cells was performed as above and analyzed with N-CoR (C-20) antibody.

### Immunofluorescence Staining

Immunofluorescence staining was carried out as described previously [13], [14]. Briefly, cells were cytospun onto glass slides, fixed with 3% paraformaldehyde and permeabilized with 0.2% Triton-X-100. After blocking, cells were stained with primary antibody followed by staining with fluorescence labeled secondary antibodies. The cell nuclei were stained with 4′,6-diamidino-2-phenylindole (DAPI) (Sigma Aldrich, MO, USA). Slides were visualized using confocal microscopy.

### Human Phospho-kinase Array

Identification of activated kinases was performed using the Proteome Profiler™ Human Phospho-kinase antibody array system (R&D systems, MN, USA) according to manufacturer’s instructions. Briefly, cells were lysed and lysates were applied to the provided membranes spotted with antibodies raised against the phosphorylated/activated form of a panel of kinases. Membranes were then analyzed using standard western blotting techniques. Pixel density of the activated kinases in 3 independent experiments was then determined using imaging software (Image J) and the average values were plotted as histograms.

### Treatment with Akti-X or Akt siRNA

Cells were treated for 24 hours with either vehicle or Akti-X (Merck, Darmstadt, Germany) [36], [37] before harvesting the cells for protein analysis by western blotting. For siRNA mediated Akt knockdown in THP-1 cells, siRNA against Akt 5′-ATA CCG GCA AAG AAG CGA TGC TGC A-3′ (Qiagen, Hilden, Germany) was synthesized as fully annealed oligonucleotide duplexes [38]. Cells were transfected with siRNA by electroporation using the Cell Line Nucleofector kit V (Amaxa, Cologne, Germany). siRNA targeting the luciferase sequence 5′-CGT ACG CGG AAT ACT TCG A-3′ was used as control.

### N-CoR Phosphorylation Assay

THP-1 cells were lysed in IP buffer (20 mM Tris pH7.4, 300 mM NaCl, 0.5% NP-40, 1 mM EDTA, 200 µM AEBSF, 1 tablet/50 ml complete protease inhibitor tablet [Roche, Germany], 1 mM NaF, 20 mM β-glycerolphosphate, 10 ul/ml phosphatase inhibitor cocktail I [Sigma, USA], 10 µl/ml phosphatase inhibitor cocktail [Sigma, USA]) and subjected to immunoprecipitation using N-CoR (C-20) antibody. Immunoprecipitated protein was analyzed by western blotting using either the pan-phospho serine/threonine antibody (Upstate technologies, NY, USA) or the phospho- Akt substrate (RXRXX pS/pT) antibody (Cell Signaling, MA, USA). For the quantification of immunoprecipitated N-CoR protein, membranes were stripped and re-probed with N-CoR antibody (C-20). For N-CoR phosphorylation by ectopic Akt, 293T cells were transfected with pAct-N-CoR-Flag and pGFP-Myr-Akt or empty vector. Cells were harvested and subjected to immunoprecipitation by Flag antibody (Sigma, CA, USA) and analyzed by western blotting as described above.

### *In vitro* Phosphorylation Assay

Flag-tagged N-CoR from 293T cells transfected with pAct-N-CoR-Flag was immunoprecipitated with affinity Flag M2 resin (Sigma Aldrich, MO, USA). Purified flag-tagged N-CoR was then eluted from the resin using 3× flag peptide (Sigma Aldrich, MO, USA). Active Akt kinase was purified from pGFP-Myr-Akt transfected 293T cells using anti-pAkt (Ser473) immobilized beads (Cell Signaling Technologies, CA, USA) and the phosphorylation assay was performed using the non-radioactive Akt kinase assay kit (Cell Signaling Technologies, CA, USA) as describe by the manufacturer. Briefly, purified active Akt kinase was re-suspended in 50 µl of 1× kinase buffer supplemented with 1 µl of 10 mM and 4 µg purified flag-tagged N-CoR protein. The mixture was incubated at 30°C for 30 minutes for the phosphorylation to take place. The reaction was then terminated with 3× SDS sample buffer. *In vitro* phosphorylation of N-CoR was then analyzed by western blotting with phospho Akt substrate (RXRXX pS/pT) antibody. Amount of N-CoR added to the reaction was detected using anti-Flag antibody while Akt kinase was detected using the phospho-Akt (Ser 473) antibody.

### Site-directed Mutagenesis and RT-PCR Analysis

To generate the N-CoR mutants S1450A, T1925A and S1450E, site-directed mutagenesis was performed using the GeneTailor site-directed mutagenesis system (Invitrogen, Carlsbad, CA, USA). All sequences of the mutant constructs were confirmed by sequence analysis. The sequences of forward and reverse primers used in the mutagenesis and subsequent sequencing analysis are appended in Table S1 and Table S2. Total RNA was isolated using RNeasy® Mini Kit (Qiagen GmBH, Hilden, Germany). Converted cDNA was subjected to RT-PCR analysis using Accuprime Taq polymerase system (Invitrogen, Carlsbad, CA, USA). The sequence of the primers is presented in Table S3.

### Dual Luciferase Reporter Assay

293T was co-transfected with 1 µg of full-length promoter/firefly luciferase reporter plasmid or promoter-less pGL3-basic vector, 5 ng of CMV/renilla luciferase plasmid and various dosages of pAct-Flag/N-CoR (WT) or S1450E, using Lipofectamine 2000 (Invitrogen, Carlsbad, CA, USA). The cells were harvested and reporter activity determined 72 hrs post-transfection.

### Determination of Cell Proliferation

Cell proliferation assay was carried out using the CellTiter 96® AQ_ueous_ One Solution Cell Proliferation Assay kit [3-(4,5-dimethylthiazol-2-yl)-5-(3 carboxymethoxyphenyl)-2-(4-sulfophenyl)-2H-tetrazolium, inner salt; MTS] (Promega, WI, USA) as described by the manufacturer. Cells were treated with Akti-X in a dose dependent manner for 72 hours. The spectrophotometric absorbance was measured using a microplate reader (Ultramark, Biorad, CA, USA) at wavelength 490 nm with a reference wavelength of 655 nm.

### Statistical Analysis

The results of the proliferation assays were reported as mean ± SD. Statistical analysis was performed using unpaired t-test. P value less than 0.05 was considered to be statistically significant.

## Results

### Misfolded Conformation Dependent Loss (MCDL) of N-CoR in AML-M5

**Figure 1:**
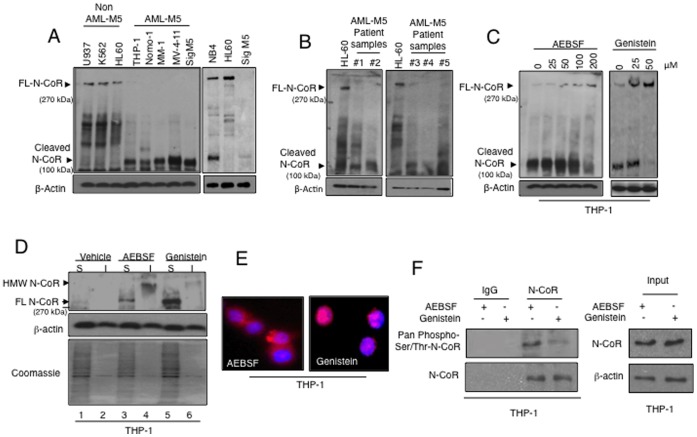
Misfolded conformation dependent loss (MCDL) of N-CoR in AML-M5. *A*, An aliquot of whole cell extract of various AML derived cells as mentioned on the top of each lane was resolved in SDS-PAGE and stained with N-CoR antibody. *B*, An aliquot of whole cell extract of AML-M5 patient samples was resolved in SDS-PAGE and stained with N-CoR antibody. N-CoR level in HL-60 was used as positive control. *C*, N-CoR is stabilized by AEBSF and genistein. Level of full length and cleaved N-CoR protein in THP-1 cells treated with AEBSF or genistein in a dose dependent manner was determined by western blotting assay using N-CoR antibody. *D*, Native N-CoR conformation is rescued by genistein but not by AEBSF. Relative solubility/insolubility of N-CoR protein in AEBSF or genistein treated THP-1 cells was determined by protein solubility assay. Soluble (S) and insoluble (I) fractions of AEBSF- or genistein-treated THP-1 cells were separated by high speed centrifugation and N-CoR level in each fraction was determined by western blotting assay using N-CoR antibody. A HMW (high molecular weight) variant of N-CoR protein, which was part of the insoluble fraction, was detected only in AEBSF treated cells. The relative solubility/insolubility of β-actin in each fraction was used as a control. The level of total of protein in each fraction was determined by coomassie blue staining. *E*, Subcellular distribution of N-CoR (red signal) in THP-1 cells treated with AEBSF (200 µM) or genistein (50 µM) was determined by confocal microscopy. DNA was stained with DAPI (blue signal). *F*, Level of serine/threonine phosphorylated N-CoR (upper panel) or total N-CoR (lower panel) in THP-1 cells treated with AEBSF or genistein was determined by staining the immunoprecipitated (IP) N-CoR with a generic phospho serine/threonine antibody or N-CoR antibody respectively. N-CoR protein level in each treated sample used in IP assay was determined by western blotting (right panel).

Given the key role of N-CoR in the transcriptional repression of *Flt3* [11], we hypothesized that de-regulation of N-CoR mediated transcriptional control as a result of its misfolding might have a significant implication in the pathogenesis of AML. Therefore, to characterize the misfolding status of N-CoR in various AML subtypes, we first determined the level of natively folded or stable (full length) N-CoR in various AML and non-AML cells through western blotting assay. In contrast to natively folded N-CoR which migrates as a 270 kDa band in western blotting assay, the misfolded N-CoR is evidently unstable and usually appears as a cleaved 100 kDa fragment in the same assay [11]–[14]. When analyzed by western blotting assay, no intact (270 kDa) N-CoR was detected in various AML-M5 derived cell lines; though an intact N-CoR of 270 kDa was evidently presented in all three non-AML-M5 derived cell lines U937, K562 and HL-60 used as controls (Fig. 1A). Instead of full length N-CoR, all AML-M5 derived cell lines contained a cleaved N-CoR fragment of 100 kDa (Fig. 1A). The absence or loss of full length N-CoR in AML-M5 cells was not due to a lack of expression of N-CoR mRNA, as the level of N-CoR transcript in all five AML-M5 derived cells was more or less comparable to that of non-AML-M5 derived cell lines (Fig. S1). Interestingly, N-CoR displayed similar pattern of processing in multiple primary human AML-M5 patient samples, suggesting that N-CoR processing was clinically relevant and not an artifact of cell line system (Fig. 1B).

The loss of N-CoR observed in AML-M5 cells largely resembled the pattern of N-CoR loss previously reported by us in APL [12], [13], [39]. The N-CoR loss in APL was essentially triggered by PML-RARα-induced phosphorylation of N-CoR, altering its conformation to an extent where it became unstable and eventually degraded by a protease-based protein quality control mechanism. The protease inhibitor AEBSF and the kinase inhibitor genistein effectively blocked the loss of N-CoR in APL [12]–[14]. While AEBSF and genistein abrogated the loss of misfolded N-CoR in APL with equal effienciy, the undelying mechanisms were quite different. The stabilization of N-CoR by genistein largely resulted from the restoration of misfolded N-CoR to its native conformation, while AEBSF-induced stabilzation of N-CoR simply resulted from the inhibition of the protease involved in the degradation of misfolded N-CoR protein. To investigate whether N-CoR loss in AML-M5 cells was also triggered by an APL like phosphorylation-induced misfolding, we first tested the effects of AEBSF or genistein on the status of N-CoR in THP-1 cells, a representative cell line of AML-M5. Both AEBSF and genistein effectively stabilized the full length N-CoR protein in THP-1 cells in a dose dependent manner (Fig. 1C). Besides, AEBSF treatment caused the accumulation of a high molecular weight (HMW) detergent insoluble N-CoR protein band; while N-CoR stabilized by genistein was largely soluble (Fig. 1D). While genistein caused a significant increase in the level of nuclear N-CoR, no such increment was observed after AEBSF treatment (Fig. 1E). While AEBSF treatment led to an amplification of ER stress as manifested by the accumulation of HMW form of ER stress marker PDI; genistein caused a significant reduction in ER stress level (Fig. S2). These findings collectively suggested that N-CoR which was subjected to degradation in AML-M5 cells was actually a misfolded protein.

Previously, we demonstrated that PML-RARα-induced aberrant serine/threonine phosphorylation played a key role in the misfolding of N-CoR protein in APL [14], [39]. To investigate whether N-CoR misfolding in AML-M5 was also caused by aberrant serine/threonine phosphorylation, N-CoR immunoprecipitated (IP) from the whole cell extracts of AEBSF or genistein treated THP-1 cells was probed with a generic pan-phospho serine/threonine antibody. As shown in Fig. 1F, N-CoR immunoprecipitated from the AEBSF treated THP-1 cells displayed significantly higher levels of serine/threonine phosphorylation when compared to N-CoR immunoprecipitated from genistein treated cells, suggesting that N-CoR which was subjected to degradation in THP-1 cells was phosphorylated.

### Identification of Akt as the Potential Kinase Responsible for N-CoR Misfolding

**Figure 2:**
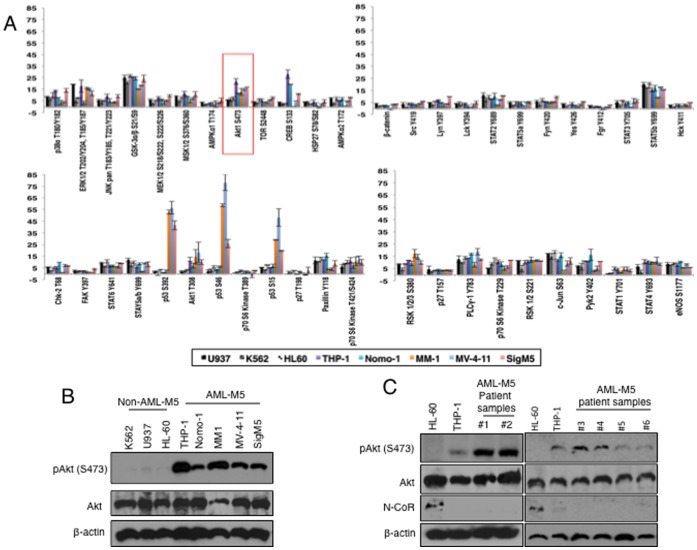
Selective activation of Akt in AML-M5 cells. *A,* Selective activation of Akt kinase in AML-M5 cells. Level of activated kinases in five AML-M5 (indicated by solid color bars) and three non-AML-M5 (HL-60, U937 and K562- indicated by patterned black and white bars) derived cell lines were determined by screening the human phospho-kinase antibody array (Proteome Profiler™) with whole cell extract of each cell mentioned above. Activity of each kinase available in the array, in these two subsets of cells was quantified by densitometric analysis of all available kinases in the human phospho-kinase array. Relative pixel densities are represented as bar graphs. Results are averages of 3 independent experiments. Only Akt kinase activity, as indicated by the phosphorylation of the Ser473 residue, is selectively upregulated in all N-CoR null AML-M5 derived cell lines as marked by a box. *B*, Level of activated Akt (pAkt-Ser473) in various AML-M5 and non-AML-M5 derived cell lines were determined by western blotting. *C*, Level of activated Akt (pAkt-Ser473) and N-CoR protein in six human AML-M5 patient samples were determined by western blotting.

Next, to identify the kinase involved in N-CoR phosphorylation, we screened a human phospho-kinase antibody array (Proteome Profiler™) with the extracts of five AML-M5 (THP-1, Nomo-1, MM1, MV-4–11 and SigM5) and three non-AML-M5 (U937, HL-60 and K562) derived cell lines. Among all the kinases analyzed, only Akt was found to be activated (through phosphorylation at serine 473) in all five AML-M5 derived cell lines when compared to three non-AML-M5 derived cell lines (Fig. 2A, Fig. S3, Table S4). Consistent with the findings of the antibody array, the level of phosphorylated Akt (Serine 473) in western blotting assay was also significantly higher in all AML-M5 derived cell lines (Fig. 2B) as well as in multiple AML-M5 patient samples in which N-CoR was lost (Fig. 2C). Serine 473 phosphorylation of Akt is crucial for its growth promoting function [40]–[43], and Akt was routinely found to be serine 473 phosphorylated in primary human AML-M5 cells [18].

**Figure 3:**
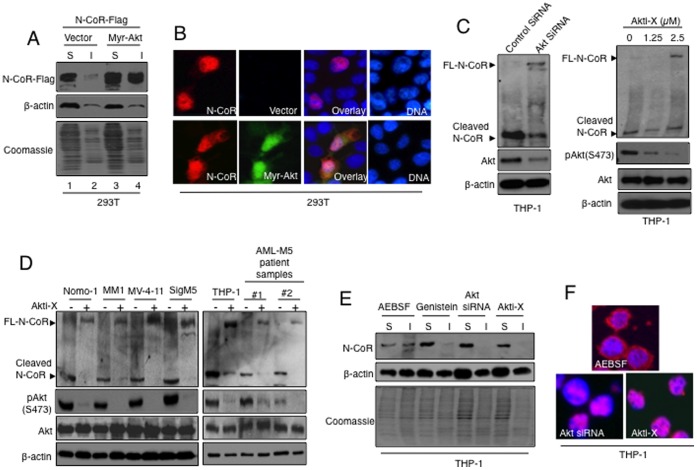
Akt promotes N-CoR misfolding. *A,* Relative solubility/insolubility of flag tagged N-CoR expressed with the constitutively active Akt (myr-Akt) in 293T cells was determined by protein solubility assay. Soluble (S) and insoluble (I) fractions were separated by high-speed centrifugation and N-CoR levels in each fraction were determined by western blotting assay using Flag antibody. The constitutively active Akt (myr-Akt) caused a significant increase in insoluble N-CoR protein levels. The relative solubility/insolubility of β-actin in each fraction served as a control. The level of total protein in each fraction was determined by coomassie blue staining. *B,* Subcellular distribution of N-CoR-Flag (red fluorescence) in 293T cells transfected with flag-tagged N-CoR with or without the constitutively active GFP tagged myr-Akt (green fluorescence) was determined by confocal microscopy. N-CoR-Flag was targeted to the cytosol when co-expressed with GFP-myr-Akt, while N-CoR-Flag expressed with empty vector was localized mainly in the nucleus. *C,* Inhibition of Akt leads to N-CoR stabilization. N-CoR and Akt level in THP- 1 cells treated with Akt or control siRNA was determined by western blotting with the respective antibodies (left panel). Levels of N-CoR, pAkt and Akt in THP-1 cells treated in a dose dependent manner with Akti-X, the commercially available specific inhibitor of Akt, was similarly determined (right panel). *D,* Therapeutic inhibition of Akt leads to N-CoR stabilization in primary and secondary AML-M5 cells. Levels of N-CoR, pAkt and Akt in AML-M5 cells (left panel) and human primary AML-M5 cells (right panel) treated in a dose dependent manner with Akti-X was determined. *E*, Native N-CoR conformation is rescued by Akt abrogation. Relative solubility/insolubility of N-CoR protein in Akt siRNA or Akti-X treated THP-1 cells was determined by protein solubility assay. Soluble (S) and insoluble (I) fractions of treated THP-1 cells were separated by high speed centrifugation and N-CoR level in each fraction was determined by western blotting assay using N-CoR antibody. THP-1 cells treated with AEBSF or genistein was used as controls. The relative solubility/insolubility of β-actin in each fraction served as an experimental control. The level of total of protein in each fraction was determined by coomassie blue staining. *F,* Subcellular distribution of N-CoR (red signal) in THP-1 cells treated with AEBSF at 200 µM, Akt siRNA or Akti-X at 2.5 µM was determined by confocal microscopy.

The role of Akt in N-CoR misfolding was further characterized by employing myr-Akt, a constitutively activated form of Akt in which the myristoylation signal is linked in frame to the Akt sequence [44]. The conformation of flag-tagged N-CoR co-expressed with myr-Akt in 293T cells was determined through protein solubility assay and analysis of sub-cellular distribution. When N-CoR was co-expressed with myr-Akt, the level of insoluble N-CoR protein was significantly increased, suggesting a direct role of myr-Akt in N-CoR misfolding (Fig. 3A). Consistent with the findings of the solubility assay, N-CoR co-expressed with myr-Akt displayed a predominantly cytosolic distribution pattern, suggesting that it has adopted a misfolded conformation (Fig. 3B, lower panel). To further define the role of Akt in N-CoR misfolding, the solubility and sub-cellular distribution of N-CoR in THP-1 cells was determined after Akt inhibition with siRNA or Akti-X, a commercially available selective small molecular inhibitor of Akt [36], [37]. Selective Akt inhibition in THP-1 cells by either means led to the stabilization of full length N-CoR (Fig. 3C). As observed in THP-1 cells, selective Akt inhibition with Akti-X also led to N-CoR stabilization in other AML-M5 derived cells (Fig. 3D left panel) as well as in human primary AML-M5 cells (Fig. 3D right panel). Interestingly, N-CoR stabilized by Akt siRNA or Akti-X was detergent soluble (Fig. 3E) and predominantly nuclear (Fig. 3F), suggesting that N-CoR regained its native conformation after Akt inhibition. Taken together, these observations suggested a direct role of Akt in the misfolding of N-CoR protein.

### Akt-induced Phosphorylation of N-CoR Promotes Misfolding

**Figure 4:**
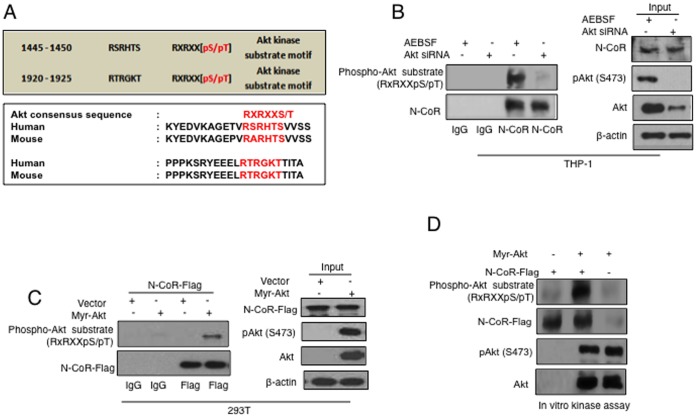
N-CoR is phosphorylated by Akt. *A*, Two potential Akt consensus motifs were identified in N-CoR. The screen capture of the results of the N-CoR putative kinase recognition motif search in the human protein reference database program is shown. Only the sequences that matched to the Akt substrate motifs in the search are presented (upper panel). The Akt substrate motif and it surrounding sequences are highly conserved in human and mouse N-CoR sequences (lower panel). *B,* Genetic ablation of Akt abrogated N-CoR phosphorylation. Level of N-CoR protein phosphorylated (upper panel, left) at the Akt consensus motif in THP-1 cells treated with AEBSF or Akt siRNA was determined by staining the immunoprecipitated (IP) full length N-CoR protein (lower panel, left) with phospho-Akt substrate (RXRXX pS/pT) antibody. The levels of N-CoR, pAkt (Ser 473) and Akt in crude cellular extracts used in IP assay were determined (right panel). *C*, Level of N-CoR protein phosphorylated (upper panel, left) at the Akt consensus site in flag-tagged N-CoR protein immunoprecipitated (lower panel, left) from 293T cells transfected with myr-Akt or control plasmid was determined by western blotting with a phospho-Akt substrate (RXRXX pS/pT) specific antibody. An aliquot of crude cell extract was probed with the respective antibodies to determine the levels of flag-N-CoR, pAkt (Ser 473) and Akt (right panel). *D,* Constitutively active Akt phosphorylates N-CoR *in vitro*. Affinity purified flag-tagged N-CoR was incubated with purified myr-Akt and the level of phosphorylated N-CoR protein after the incubation was determined by western blotting assay with the phospho-Akt substrate (RXRXX pS/pT) antibody.

Next, we sort to clarify if N-CoR was a natural substrate of Akt and if Akt promoted N-CoR misfolding by inducing its phosphorylation directly. Akt preferentially phosphorylates the serine/threonine (**S/T**) residues immediately followed by the Akt recognition motif RXRXX **(**RXRXX **S/T;** where X represents any amino acid) in its substrate. Analysis of the N-CoR open reading frame with the bioinformatics tools available at Human Protein Reference database [45] revealed the presence of two putative Akt consensus motifs in the N-CoR amino acid sequence spanning the residues 1445–1450 and 1920–1925 (Fig. 4A, upper panel). Sequence alignments performed with ClustalW revealed that these two putative Akt recognition motifs and its surrounding regions were highly conserved in the mouse and human N-CoR amino acid sequence (Fig. 4A, lower panel). These findings suggested that N-CoR could be a natural substrate of Akt. Most of the natural substrates of Akt could be recognized with an antibody raised against the phosphorylated serine or threonine residues at the Akt recognition motif RXRXX **S/T.** To confirm if N-CoR is indeed a substrate of Akt, N-CoR immunoprecipitated from mock or Akt-siRNA transfected THP-1 cells was probed with the phospho-Akt substrate (RXRXX pS/pT) antibody. As shown in Figure 4B, N-CoR immunoprecipitated from the mock transfected THP-1 cells displayed significantly higher levels of RXRXX S/T phosphorylation, which was completely abrogated after Akt knockdown, suggesting a crucial role of Akt in N-CoR phosphorylation. Similarly, flag-tagged N-CoR displayed significantly higher levels of RXRXX S/T phosphorylation when co-expressed in 293T cells with the constitutively active myr-Akt (Fig. 4C). Moreover, affinity purified myr-Akt directly phosphorylated affinity purified flag-tagged N-CoR protein when incubated together in an *in-vitro* kinase assay (Fig. 4D). These findings clearly indicated that N-CoR was a direct substrate of Akt and Akt-induced phosphorylation of N-CoR might be crucial for its misfolding.

### Akt-induced Phosphorylation of N-CoR at Serine 1450 is Essential for Misfolding

**Figure 5:**
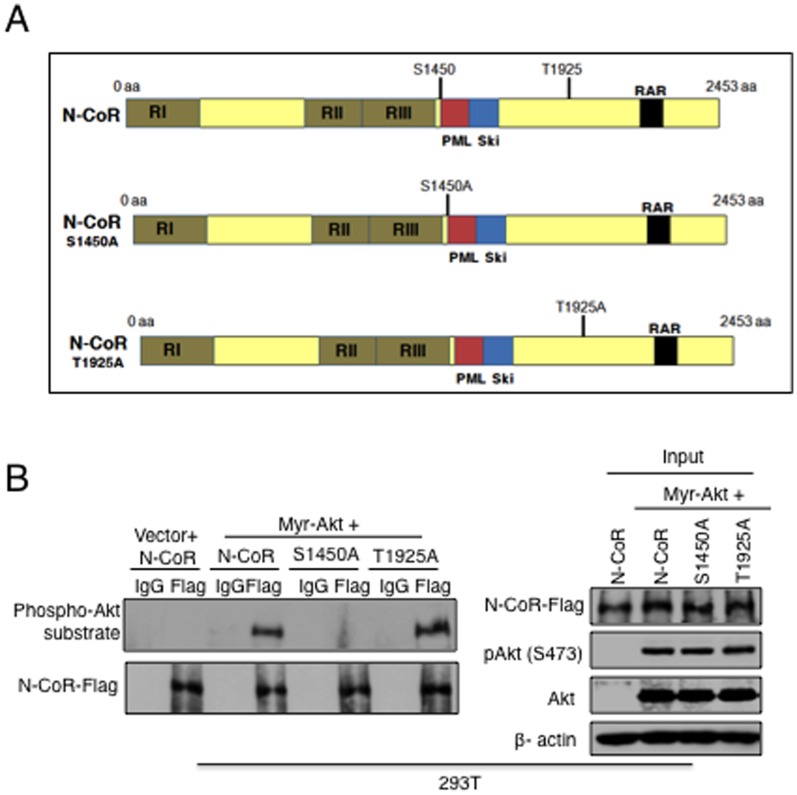
Akt promotes N-CoR phosphorylation at serine 1450. Serine to Alanine substitution at position 1450 completely abrogated the Akt-induced N-CoR phosphorylation. *A,* Schematic representation of various serine or threonine N-CoR mutant constructs. *B,* Level of phosphorylated N-CoR (upper panel) in total N-CoR protein immunoprecipitated (lower panel) with Flag antibody from 293T cells transfected with flag-tagged WT or mutant (S1450A and T1925A) N-CoR expression plasmids with or without constitutively active Akt (myr-Akt) was determined with phospho-Akt substrate (RXRXX pS/pT) antibody. To determine the levels of WT and mutant N-CoR proteins, an aliquot of whole cell extract used in the immunoprecipitation assay described above was probed with flag antibody (right panel). Levels of pAkt (Ser 473) and Akt in whole cell extract were probed with the respective antibodies (right panel).

Next to decipher which of the two putative Akt consensus motifs of N-CoR described above (Figure 4A) was actually phosphorylated by Akt, we generated two flag-tagged N-CoR mutants namely S1450A and T1925A in which either the serine 1450 or threonine 1925 residues was replaced with a non-phosphorable alanine residue (Figure 5A, Fig. S4). The ability of these mutants to be phosphorylated by myr-Akt was determined in 293T cells by measuring their RXRXX S/T phosphorylation levels in immunoprecipitation and western blotting assays. As shown in Figure 5B (left panel), the N-CoR S1450A mutant displayed complete abrogation of phosphorylation by myr-Akt, while the N-CoR T1925A mutant could still be phosphorylated at the same level; suggesting that serine 1450 was the actual phosphor-acceptor site. This finding suggested that Akt directly phosphorylated N-CoR at serine 1450.

**Figure 6:**
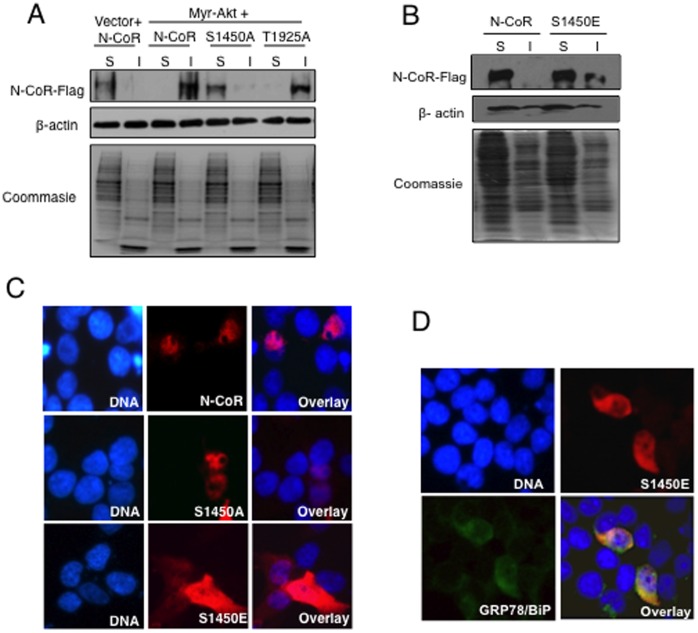
Serine 1450 phosphorylation is critical for Akt-induced N-CoR misfolding. *A*, Serine to alanine substitution at 1450 abrogated Akt-induced N-CoR misfolding. Relative solubility/insolubility of flag-tagged N-CoR (WT and S1450A or T1925A mutants) in 293T cells transfected with the constitutively active myr-Akt was determined by protein solubility assay. Soluble (S) and insoluble (I) fractions were separated by high-speed centrifugation and N-CoR levels in each fraction were determined with flag antibody (upper panel). The relative solubility/insolubility of β-actin in each fraction was determined as a control (middle panel). The level of total of protein in each fraction was determined by coomassie blue staining (lower panel). *B,* N-CoR S1450E, the phosphomimetic mutant of N-CoR, displayed signs of misfolding. Relative solubility/insolubility of flag-tagged N-CoR (WT and S1450E) in 293T cells was determined by protein solubility assay. *C,* S1450E is localized in cytosol. Subcellular distribution of flag-tagged N-CoR (WT, S1450A and S1450E) was determined by confocal microscopy after staining of the cells transfected with each plasmid with flag antibody. *D,* S1450E can induce ER stress. Level of GRP78/BiP (green) in 293T cells transfected with S1450E (red) was determined by confocal microscopy after staining the cells with flag and GRP78/BiP antibodies.

Next, to investigate the link between serine 1450 phosphorylation and misfolding, the effect of myr-Akt on the misfolding of N-CoR S1450A or N-CoR T1925A was analyzed in 293T cells transfected with myr-Akt and flag tagged N-CoR S1450A or N-CoR T1925A plasmids. While myr-Akt could effectively render the N-CoR T1925A mutant insoluble, the N-CoR S1450A mutant remained largely soluble (Fig. 6A), suggesting a crucial role of serine 1450 in Akt-induced misfolding of N-CoR. Phosphorylation usually triggers a qualitative change in protein conformation by conferring a negative charge to the residue it phosphorylates. Therefore, to further confirm that the Akt-induced N-CoR phosphorylation at serine 1450 actually triggers the misfolding of N-CoR protein, we tested the solubility and subcellular distribution of N-CoR S1450E, a phosphomimic mutant of N-CoR in which the serine (S) at 1450 was replaced with glutamic acid (E) that carries a native negative charge due to its -COOH group (Fig. S5). When expressed in 293T cells, a significant portion of N-CoR S1450E was accumulated in the insoluble fraction and was predominantly localized in the cytosol (Fig. 6B & C), suggesting that N-CoR S1450E mutant could spontaneously assume a misfolded conformation. Like the original misfolded N-CoR, N-CoR S1450E could also induce ER stress (a key impact of misfolded protein) as evidenced by the up regulation of a key ER stress marker GRP78/BiP in cells expressing N-CoR S1450E (Fig. 6D). Collectively, these findings suggested that Akt-induced phosphorylation of N-CoR at serine 1450 triggers a conformational change in N-CoR leading to its misfolding and eventual loss in AML-M5.

**Figure 7:**
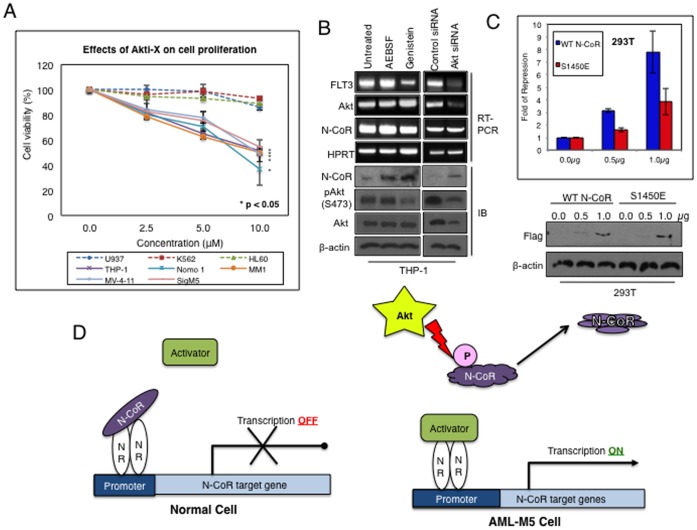
Role of Akt in the growth of AML-M5 cells and in the de-repression of N-CoR target gene. *A,* Akt is linked to the growth of AML-M5 cells. Growth of various AML-M5 (THP-1, Nomo-1, MM-1, MV-4–11 and SigM5) and non-AML-M5 (U937, K562 and HL-60) cells treated with Akti-X, a selective inhibitor of Akt, was determined. Asterisk indicates p<0.05. *B*, Akt inhibition restores N-CoR mediated *Flt3* gene repression. Level of *Flt3* transcripts in THP-1 cells treated with AEBSF, genistein or Akt siRNA was determined by RT-PCR. Transcript and protein levels of N-CoR, Akt and pAkt (Ser473) were also determined. *C,* Loss of N-CoR mediated repression of *Flt3* due to misfolding. The effect of wild type N-CoR (WT) or the spontaneously misfolded N-CoR (phosphomimetic S1450E) on the *Flt3* promoter was determined by luciferase assay (top panel). The amount of N-CoR transfected was determined by western blotting assay with flag antibody (bottom panel). *D,* Schematic representation of transcriptional control imparted by native and misfolded N-CoR. During cellular differentiation, the N-CoR containing co-repressor complex occupies the promoter of growth promoting N-CoR target genes such as *Flt3,* effectively blocking their expression (left panel). In AML-M5 cells, misfolded conformational dependent loss of N-CoR due to Akt mediated phosphorylation results in the dissociation of the co-repressor complex from the promoter of N-CoR target genes, thus allowing the transcriptional activators to access the promoter and activate the gene (right panel).

To define the role of Akt in the growth of AML-M5 cells, we next investigated the effect of Akti-X on the growth of AML-M5 cells. Akti-X inhibited the growth of multiple AML-M5 derived cells in a dose dependent manner while its effect on non-AML-M5 cells was minimal (Fig. 7A). As shown by us recently, the growth promoting effect of misfolded N-CoR is partly mediated by the de-repression of *Flt3*, a transcriptional target of N-CoR [11]. Therefore, to test whether Akt promoted the growth of AML-M5 cells through *Flt3* de-repression, level of *Flt3* transcript in THP-1 cells was measured after siRNA-mediated Akt ablation. As shown in Figure 7B, Akt ablation led to a significant down regulation of *Flt3* level in THP-1 cells, possibly through the restoration of native N-CoR conformation and function as observed with genistein (Fig. 7B, left panel). Moreover, the constitutively misfolded phosphomimetic mutant of N-CoR, N-CoR S1450E, could not repress the luciferase reporter activity driven by *Flt3* promoter as efficiently as the wild type N-CoR did (Fig. 7C, left panel), suggesting a link between N-CoR phosphorylation and loss of N-CoR’s repressor function. These results suggest that Akt-induced phosphorylation of N-CoR compromised its function as a transcriptional repressor, leading to the ectopic reactivation of growth promoting genes like *Flt3*, ultimately contributing to malignant growth and transformation of AML-M5 cells (Fig. 7D).

## Discussion

The phosphorylation of N-CoR by Akt at serine 1450 may have destabilized its core by altering the local free energy, eventually leading to its misfolding and subsequent loss in AML-M5 cells. The misfolding of N-CoR could also expose its hydrophobic residues that are normally buried in the core when N-CoR is in its native conformation, and facilitate the non-functional associations of N-CoR with random proteins while blocking the functional interactions with bona fide cellular partners. Moreover, exposure of hydrophobic residues in the misfolded conformation may facilitate its recognition and targeting by the cellular protein quality control machinery and molecular chaperones, leading to N-CoR’s degradation and eventual loss in AML-M5 cells. The ER targeting of misfolded N-CoR could initially trigger ER stress; however, the eventual degradation of misfolded N-CoR by the protein quality control machinery may lead to the attenuation of ER stress, ultimately protecting the AML-M5 cells from ER stress-induced apoptosis. Interestingly, an important role of Akt in the attenuation of ER stress and protection of cells from ER stress-induced apoptosis has recently been identified [46], [47].

AML-M5 is caused primarily by a wide array of genetic defects, including chromosomal translocations involving multiple genes. However, the leukemic cells in all AML-M5 variants display an almost identical phenotype, characterized by the differentiation arrest of myeloid cells coupled with the ectopic reactivation of cellular self-renewal and growth potentials. Despite the progress made in the identification and characterization of the diverse genetic footprint of AML-M5, how exactly do these diverse genetic anomalies create an indistinguishable and almost uniform cellular and clinical feature in the different variants of AML-M5 is not known. It is likely that these diverse genetic anomalies promote the transformation of AML-M5 cells by targeting a common transcriptional co-factor like N-CoR which is essential for the normal growth and maturation of early myeloid cells.

Genetic or post-translational aberrations are linked to loss of function phenotype in many tumor suppressor proteins such as p53 [48]–[50], WT1 [51]–[53], VHL [54], [55] and Merlin [56]. These aberrations often alter the conformation of proteins, leading to the production of misfolded proteins. We have previously shown how aberrant post-translational modification altered the conformation of N-CoR in APL [12]–[14] and NSCLC [57], thereby compromising its function. In this report, we delineated the events underlying the misfolding of N-CoR and have identified the kinase which contributes to N-CoR misfolding in AML-M5. The identification of Akt as the key inducer of N-CoR misfolding links the oncogenic Akt signaling pathway to the N-CoR misfolding pathway. The N-CoR misfolding pathway described in this report could be the common downstream thread of pleiotropic Akt signaling activated by various forms of oncogenic insults in some subtypes of leukemia and solid tumors.

## Supporting Information

Figure S1 **Relative expression of N-CoR transcript levels in HL-60, U937 and AML-M5 cell lines as determined via RT-PCR (A) and (B) Real time PCR analysis.** No significant differences in transcript levels were observed.
(TIFF)

Figure S2 **A, AEBSF induced N-CoR stabilization results in the amplification of ER stress as evidenced by the upregulation of HMW PDI expression.**
(TIFF)

Figure S3 **Raw blots of the Human Kinase Array used for quantification of kinase hyper-activation.** Coordinates of each spot on the blot are labeled. Pixel quantification is carried out after normalization with positive and loading controls using ImageJ. Full list of kinases represented by each coordinate is depicted in Table S4.
(TIFF)

Figure S4 **Sequencing chromatograms of successful Serine-Alanine 1450 (S1450A) and Threonine-Alanine 1925 (T1925A) mutants.** A box on the sequence indicates desired mutations.
(TIFF)

Figure S5 **Sequencing chromatogram of successful Serine-Glutamic Acid 1450 (S1450E) mutant. A box on the sequence indicates desired mutation.**
(TIFF)

Table S1 (DOCX)

Table S2 (DOCX)

Table S3 (DOCX)

Table S4 (DOCX)

Methods S1 (DOCX)
