# Supplementary material for: Akt-Induced Phosphorylation of N-CoR at Serine 1450 Contributes to Its Misfolded Conformational Dependent Loss (MCDL) in Acute Myeloid Leukemia of the M5 Subtype
Source: PLoS One. 2013 Aug 5;8(8):e70891. doi: 10.1371/journal.pone.0070891 (PMC3733915; doi:10.1371/journal.pone.0070891)
Supplement: Table S1 — (DOCX) [file pone.0070891.s006.docx]

**Supplemental Table T1.**

**Supplemental Table T1: List of primers used in Site Directed Mutagenesis**

|  | **Forward primer** | **Reverse Primer** |
| --- | --- | --- |
| Ser1450-Ala | 5’-TGCGAGCCCGGCACACG  GCAGTGGTGAGCT-3’ | 5’-CGTGTGCCGGGCTCG CACTGGCTCGCCTG-3’ |
| Ser1450-Glu | 5’-TGCGAGCCCGGCACACG  GAAGTGGTGAGCTCT-3’ |  |
