# Supplementary material for: Akt-Induced Phosphorylation of N-CoR at Serine 1450 Contributes to Its Misfolded Conformational Dependent Loss (MCDL) in Acute Myeloid Leukemia of the M5 Subtype
Source: PLoS One. 2013 Aug 5;8(8):e70891. doi: 10.1371/journal.pone.0070891 (PMC3733915; doi:10.1371/journal.pone.0070891)
Supplement: Table S2 — (DOCX) [file pone.0070891.s007.docx]

**Supplemental Table T2.**

**Supplemental Table 2: Primers used for analysis of base pair mutations**

|  | **Forward primer** | **Reverse Primer** |
| --- | --- | --- |
| N-CoR MutA | 5’-GACAACAACTC  AGGTTCAATCAG -3’ | 5’-CTCTGGATATGG  TGTTCTGGTAG -3’ |
