# Supplementary material for: Akt-Induced Phosphorylation of N-CoR at Serine 1450 Contributes to Its Misfolded Conformational Dependent Loss (MCDL) in Acute Myeloid Leukemia of the M5 Subtype
Source: PLoS One. 2013 Aug 5;8(8):e70891. doi: 10.1371/journal.pone.0070891 (PMC3733915; doi:10.1371/journal.pone.0070891)
Supplement: Table S3 — (DOCX) [file pone.0070891.s008.docx]

**Supplemental Table T3.**

**Supplemental Table T3: List of RT-PCR primers**

| **Gene** | **Sequence** | **Annealing Temperature (^0^C)** | **Cycles** |
| --- | --- | --- | --- |
| Flt3 | Forward: 5’-TCAGGGGCAATGCCCGTCTG-3’  Reverse: 5’-CTGCATCTGCCAGCTGACATCC-3’ | 60 | 30/40 |
| N-CoR1 | Forward: 5’-GACTCTGATATGGCAGCTGCTCAG-3’  Reverse: 5’-GCTGAGCATCCGCATAGTCAGAG-3’ | 60 | 30 |
| HPRT (house keeping) | Forward: 5’-GAAGGAGATGGGAGGCCATCAC-3’  Reverse: 5’-CAACAATCCGCCCAAAGGGAAC-3’ | 60 | 30 |
|  |  |  |  |
