# Supplementary material for: Akt-Induced Phosphorylation of N-CoR at Serine 1450 Contributes to Its Misfolded Conformational Dependent Loss (MCDL) in Acute Myeloid Leukemia of the M5 Subtype
Source: PLoS One. 2013 Aug 5;8(8):e70891. doi: 10.1371/journal.pone.0070891 (PMC3733915; doi:10.1371/journal.pone.0070891)
Supplement: Table S4 — (DOCX) [file pone.0070891.s009.docx]

**Supplemental Table T4:**

**Supplemental Table 4: List of kinase and their coordinates on the Human Phospho-Array Blots.**

**
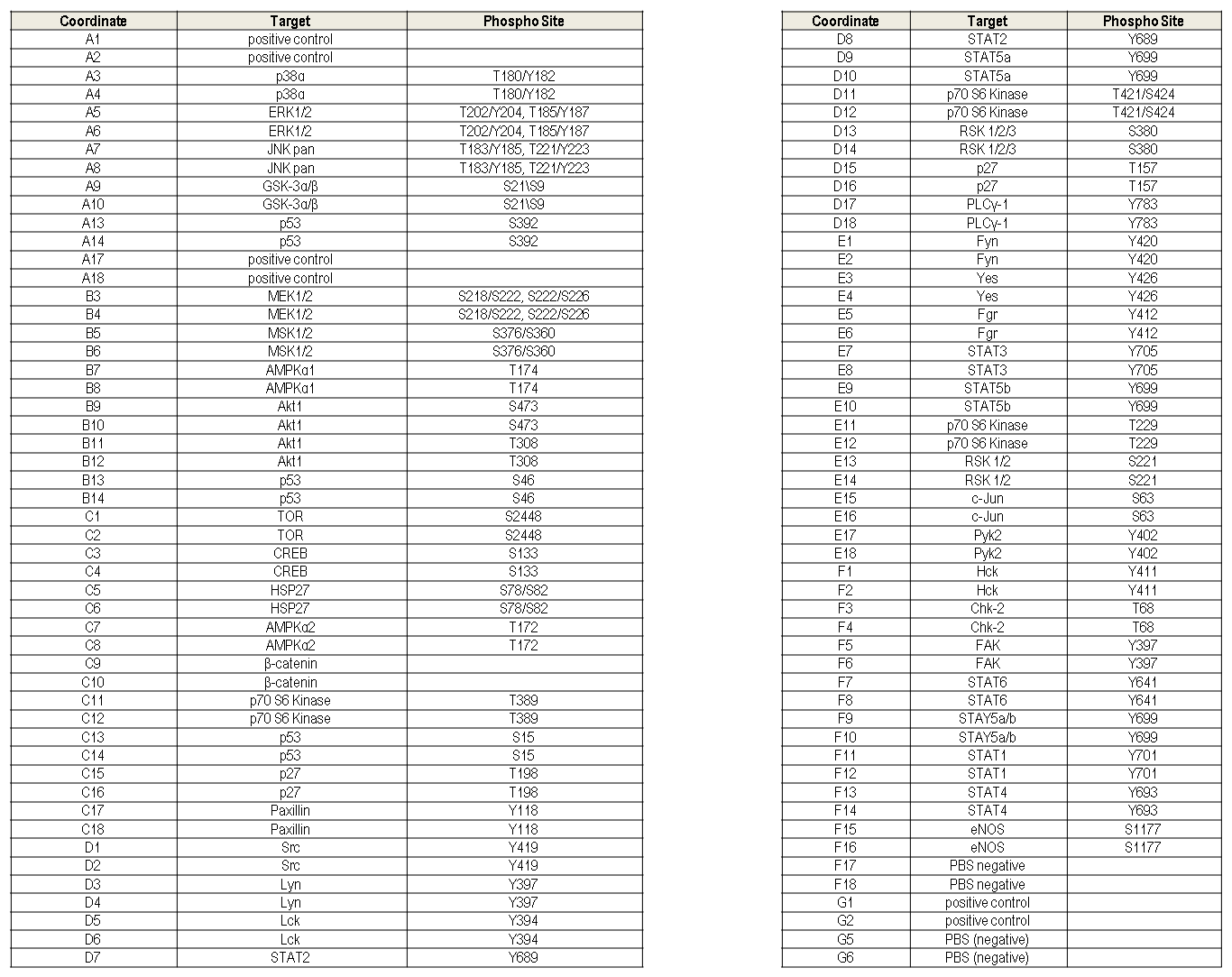
**
