# Supplementary material for: Akt-Induced Phosphorylation of N-CoR at Serine 1450 Contributes to Its Misfolded Conformational Dependent Loss (MCDL) in Acute Myeloid Leukemia of the M5 Subtype
Source: PLoS One. 2013 Aug 5;8(8):e70891. doi: 10.1371/journal.pone.0070891 (PMC3733915; doi:10.1371/journal.pone.0070891)
Supplement: Methods S1 — (DOCX) [file pone.0070891.s010.docx]

**Supplementary Materials and Methods.**

***Real time PCR assay.***

For cell lines, total RNA was isolated using the RNeasy Mini Kit (Qiagen GmBH, Hilden, Germany). From each sample, 2 µg of RNA was converted into cDNA by oligo (dT)_18_-primed reverse transcription using SuperScript II RT First-Strand kit (Invitrogen, Carlsbad, CA, USA) as described by the manufacturer. Real time PCR analysis was carried out using the Taqman® Gene Expression Assay System (Applied Biosystems, CA,USA) and C_t_ values were recorded using the ABI Prism 7300 Real Time PCR system (Applied Biosystems, CA, USA).

**Analysis of Real-Time PCR data.**

*D*ata was analyzed using the comparative C_t_ method where HL-60 cell was used as the reference sample and the HPRT gene was used as the endogenous gene control. Data representation for gene expression analysis is in the form of a bar graph. Data represented is the average obtained from three independent experiments.
